# Gut dysbiosis and vitamin-dependent immune regulation in degenerative musculoskeletal and bone diseases

**DOI:** 10.3389/fimmu.2026.1920962

**Published:** 2026-07-30

**Authors:** Nicola Stefanelli

**Affiliations:** Independent Researcher, Montecatini Terme, Italy

**Keywords:** bone remodeling, dysbiosis, gut microbiota, immune regulation, menaquinones, osteoimmunology, vitamin D, vitamin K2

## Abstract

Degenerative musculoskeletal and metabolic bone diseases are increasingly recognized as conditions sustained not only by endocrine and mechanical factors, but also by chronic low-grade immune activation and osteo-immune imbalance. This Perspective proposes a mechanistic framework in which gut dysbiosis may contribute to skeletal degeneration through alterations in vitamin-dependent immune regulation, with particular attention to the interaction between vitamin D signaling and microbiota-derived menaquinones. Dysbiosis may impair intestinal barrier integrity and increase exposure to microbial-associated molecular patterns, thereby sustaining innate and adaptive immune activation and promoting a pro-inflammatory cytokine milieu involving IL-6, TNF-α, IL-17, and IL-1β. These pathways may promote osteoclastogenesis and disrupt bone remodeling through the RANKL/RANK/OPG axis. While the immunomodulatory role of vitamin D is well established, microbiota-derived menaquinones may represent a less explored but biologically plausible interface between microbial metabolism, inflammatory signaling, and skeletal homeostasis . However, the extent to which microbiota-derived menaquinones significantly contribute to systemic vitamin K status in humans remains controversial and incompletely characterized. Within this framework, dietary patterns are conceptualized as modulators of microbial ecology and immune activation, while microbiota-modulating strategies may indirectly influence osteo-immune balance through immune-mediated mechanisms. This Perspective integrates microbial, immunological, and vitamin-dependent pathways into an immunology-centered model of skeletal degeneration and highlights the need for studies combining microbiome profiling, immune phenotyping, vitamin-dependent signaling, and bone remodeling outcomes.

## Introduction

The gut microbiota is a dynamic ecosystem involved in metabolic regulation, intestinal barrier integrity, immune signaling, and host homeostasis (1, 2). Beyond its classical gastrointestinal role, the microbiota is increasingly recognized as a potential systemic regulator capable of influencing endocrine, neurological, cardiovascular, and skeletal physiology (1–3). Growing attention has therefore been directed toward the gut-bone axis, a conceptual framework describing the interaction between microbial communities, inflammatory signaling, immune pathways, and bone remodeling (1–3).

Degenerative musculoskeletal and metabolic bone diseases, including osteoporosis, osteopenia, and age-related skeletal fragility, have traditionally been interpreted through endocrine, nutritional, and mechanical paradigms. Calcium homeostasis, hormonal regulation, aging, and physical loading remain central determinants of skeletal integrity. However, osteoimmunology has progressively demonstrated that skeletal homeostasis is also deeply interconnected with immune regulation and inflammatory signaling (4, 13).

Within this framework, chronic low-grade inflammation has emerged as a relevant contributor to skeletal degeneration. The concept of inflammaging describes the persistent activation of inflammatory pathways accompanying aging and contributing to tissue dysfunction and frailty (14). Cytokines such as IL-6, TNF-α, IL-1β, and IL-17 may sustain osteoclastogenic signaling and progressively alter the balance between bone formation and resorption (4, 13).

Gut dysbiosis may represent one contributing factor within this inflammatory environment. Alterations in microbial diversity, metabolic activity, and ecological balance can impair intestinal barrier integrity and increase exposure to microbial-associated molecular patterns, thereby sustaining innate immune activation (12). In parallel, dysbiosis may alter the production of microbial metabolites involved in immune regulation, including short-chain fatty acids and bacterially produced vitamins (8, 16).

Among vitamin-dependent pathways, vitamin D signaling represents one of the best characterized immunomodulatory systems (5–7). Through the vitamin D receptor, vitamin D influences macrophage polarization, dendritic cell maturation, T-cell differentiation, and cytokine production (5–7). In contrast, microbiota-derived menaquinones, commonly referred to as vitamin K2 forms, remain considerably less explored in the context of immune regulation (8–11). Although menaquinones are increasingly investigated for their interaction with inflammatory pathways and immune signaling, the extent to which microbiota-derived menaquinones significantly contribute to systemic vitamin K status in humans remains controversial and incompletely characterized.

This Perspective proposes that dysbiosis-driven alterations in vitamin-dependent immune regulation may contribute to osteo-immune imbalance in degenerative musculoskeletal and metabolic bone diseases. Particular emphasis is placed on the possibility that microbiota-derived menaquinones may act as modulators of inflammatory tone within the gut-immune-bone axis. Rather than framing vitamin K2 as a direct skeletal cofactor, this Perspective aims to reposition menaquinones within an immunometabolic context integrating microbial ecology, inflammatory signaling, and bone remodeling.

## The osteo-immune axis in degenerative musculoskeletal disease

Bone remodeling is continuously regulated by the coordinated activity of osteoblasts, osteoclasts, osteocytes, endocrine pathways, and immune-derived signals (4, 13). Osteoimmunology has highlighted that the skeletal and immune systems are interconnected through bidirectional communication mediated by cytokines, growth factors, and immune cell populations (4).

Under physiological conditions, immune signaling contributes to skeletal homeostasis. In pathological contexts, however, persistent inflammatory activation may promote excessive osteoclastogenesis and impaired osteoblast activity. Pro-inflammatory cytokines including IL-6, TNF-α, IL-1β, and IL-17 are among the principal mediators involved in inflammatory bone resorption (4, 13). These cytokines influence osteoclast differentiation primarily through modulation of the RANKL/RANK/OPG axis (13).

The adaptive immune system plays an important role in this process. T helper 17 cells promote osteoclastogenesis through IL-17-mediated signaling, whereas regulatory T cells may suppress inflammatory responses and partially inhibit osteoclast differentiation (4, 12). Consequently, the balance between Th17 and Treg populations represents a critical determinant of osteo-immune homeostasis.

Innate immune pathways are also closely linked to skeletal regulation. Macrophage polarization influences tissue inflammation and repair, with M1-like macrophages promoting inflammatory cytokine production and M2-like macrophages contributing to tissue remodeling and resolution. Dendritic cells and innate immune receptors participate in inflammatory amplification through recognition of microbial-associated molecular patterns (5, 12).

Importantly, these mechanisms are not restricted to autoimmune or inflammatory arthropathies. Chronic low-grade inflammation associated with aging, obesity, metabolic dysfunction, and gut dysbiosis may sustain subtle but persistent osteoclastogenic signaling (14). This inflammatory background may progressively impair bone remodeling and contribute to degenerative musculoskeletal disease.

## Vitamin D-dependent immune regulation

Vitamin D is one of the most extensively characterized nutrient-dependent regulators of immune function (5–7). Beyond its classical role in calcium and phosphate homeostasis, vitamin D acts as a pleiotropic immunomodulatory signal capable of influencing both innate and adaptive immune responses.

The biological effects of vitamin D are primarily mediated through the vitamin D receptor, which is expressed in multiple immune cell populations, including macrophages, dendritic cells, neutrophils, T lymphocytes, and B lymphocytes (5–7). This widespread expression reinforces the concept that vitamin D signaling should not be interpreted exclusively as an endocrine mechanism, but also as an integral component of immune regulation.

In innate immunity, vitamin D signaling modulates macrophage activation, dendritic cell maturation, cytokine production, and antimicrobial responses (5–7). Activation of the vitamin D receptor may reduce excessive NF-κB activation and contribute to a more controlled inflammatory phenotype (5, 6). Vitamin D signaling also promotes antimicrobial peptide expression, including cathelicidin, thereby linking nutrient-dependent pathways with host defense mechanisms (7).

Within adaptive immunity, vitamin D influences T-cell differentiation and polarization (5–7). Active vitamin D signaling has generally been associated with suppression of excessive Th1 and Th17 responses and promotion of regulatory immune phenotypes, including regulatory T cells (5–7). Because Th17-derived cytokines are strongly involved in osteoclastogenesis and inflammatory bone resorption, alterations in vitamin D signaling may directly influence osteo-immune balance.

An additional layer of complexity derives from the fact that immune cells themselves can metabolize vitamin D. Macrophages and activated immune cells express CYP27B1, allowing local conversion of circulating 25-hydroxyvitamin D into active calcitriol (7). This autocrine and paracrine regulation enables immune cells to modulate their own inflammatory responsiveness.

Within the gut-immune-bone axis, impaired vitamin D signaling may amplify dysbiosis-associated immune activation. In this context, vitamin D may act as an immunoregulatory buffer limiting excessive inflammatory amplification.

## Integration of vitamin D and vitamin K-dependent pathways

An additional aspect worth considering within this framework is the possible interaction between vitamin D- and vitamin K-dependent pathways. These systems are traditionally discussed in the context of bone metabolism, where vitamin D regulates calcium homeostasis and vitamin K-dependent proteins require γ-carboxylation for proper biological function.

However, the interaction between these vitamin systems may extend beyond skeletal mineralization. Vitamin D signaling exerts well-established effects on macrophage activity, dendritic cell function, T-cell differentiation, and cytokine production (5–7). In contrast, vitamin K-dependent pathways have historically received less attention in immunology, despite emerging evidence suggesting potential interactions with inflammatory signaling (8–11).

Experimental and preclinical studies suggest that vitamin K-related mechanisms may be associated with NF-κB activation, cytokine production, oxidative stress responses, inflammatory gene expression, and IL-6 modulation (8–11). Experimental evidence has also suggested that menaquinones may attenuate lipopolysaccharide-induced inflammatory signaling and modulate cytokine responses in immune and stromal cells (10, 11).

From a conceptual perspective, gut dysbiosis may simultaneously affect both vitamin D-dependent immune regulation and microbiota-derived menaquinone availability. This could theoretically contribute to disruption of immunoregulatory pathways and amplification of pro-inflammatory cytokine networks.

Importantly, this Perspective does not propose a fully established synergistic mechanism between vitamin D and menaquinones in human immune physiology. Current evidence remains incomplete and largely indirect. Nevertheless, the possibility that these pathways converge within inflammatory regulation represents a biologically plausible and underexplored hypothesis deserving further investigation.

## Microbiota-derived menaquinones and immune signaling

Vitamin K includes a group of structurally related compounds sharing a common naphthoquinone backbone. Among these, phylloquinone is primarily derived from plant sources, whereas menaquinones are largely synthesized by intestinal bacteria (8, 9). Different bacterial taxa contribute to menaquinone production, and microbial composition may influence both the quantity and profile of menaquinones generated within the gut environment.

Traditionally, vitamin K has been investigated mainly in relation to coagulation and bone metabolism. However, emerging evidence suggests that vitamin K-dependent pathways may also interact with inflammatory signaling and immune regulation (8–11). This possibility is particularly interesting in the context of microbiota-derived menaquinones because it positions bacterial metabolism as a potential contributor to immune tone.

Several experimental and preclinical studies suggest that vitamin K2 may influence inflammatory pathways involving NF-κB signaling, oxidative stress regulation, cytokine production, macrophage activation, and IL-6 modulation (8–11). However, much of the available evidence derives from *in vitro* studies, animal models, or indirect observational data.

Consequently, the role of microbiota-derived menaquinones in human immune physiology remains insufficiently characterized. It is still unclear to what extent bacterially produced menaquinones contribute to systemic vitamin K pools, whether dysbiosis meaningfully alters their bioavailability, and how these changes might influence immune signaling *in vivo*.

Despite these limitations, the inclusion of menaquinones within the gut-immune-bone framework remains biologically plausible. Dysbiosis-related reductions in menaquinone-producing taxa may theoretically alter inflammatory regulation and contribute to immune imbalance in chronic low-grade inflammatory conditions (14).

### Beyond NF-κB: other possible immunoregulatory mechanisms

Beyond NF-κB modulation, menaquinones may influence immune regulation through additional mechanisms. One possible pathway involves the vitamin K-dependent proteins Gas6 and Protein S. These proteins require vitamin K-dependent γ-carboxylation to interact effectively with TAM receptors, which are involved in macrophage activity, the clearance of damaged or apoptotic cells, and the control and resolution of inflammatory responses (17). Adequate vitamin K availability could therefore support mechanisms that limit excessive immune activation. However, it is not yet known whether microbiota-derived menaquinones contribute significantly to this pathway in humans.

A second possible mechanism concerns oxidative stress and lipid peroxidation. Reduced forms of vitamin K can act as antioxidants through the FSP1-dependent vitamin K cycle and may limit ferroptosis, a form of cell death associated with membrane lipid damage (18). Because lipid peroxidation and cell damage can promote the release of inflammatory signals, this mechanism may indirectly reduce chronic inflammatory activation. Its relevance to immune cells and osteo-immune remodeling, however, remains to be established.

Experimental evidence also suggests that different forms of vitamin K may have different effects on immune cells. In murine macrophages, several vitamin K forms modulated inflammatory mediators, including cytokines, cyclooxygenase-2, nitric oxide synthase, and matrix metalloproteinases. These effects varied according to the vitamin K form and side-chain length and were not fully dependent on intracellular conversion to MK-4 (19). Therefore, findings obtained with supplemental MK-4 or MK-7 cannot automatically be extended to all menaquinones produced by the intestinal microbiota.

Menaquinones may also influence gene expression through the pregnane X receptor (PXR). MK-4 has been identified as a PXR activator, and PXR signaling is involved in intestinal barrier function, oxidative stress, inflammatory regulation, and bone metabolism (20). However, most available evidence concerns MK-4, and it remains uncertain whether longer-chain menaquinones produced by gut bacteria exert comparable effects.

Overall, these mechanisms suggest that menaquinones may influence immune regulation through vitamin K-dependent proteins, TAM receptor signaling, control of oxidative stress and ferroptosis, direct effects on macrophage inflammatory responses, and PXR-mediated transcription. Nevertheless, these pathways should currently be considered biologically plausible rather than established components of the gut-immune-bone axis, because direct evidence on microbiota-derived menaquinones in human immune regulation remains limited.

### Gut microbiota and osteoblast-related bone formation

Although most research on the gut-bone axis has focused on osteoclastogenesis and bone resorption, human studies suggest that changes in the gut microbiota may also be associated with bone formation. In postmenopausal women, differences in gut microbial composition and microbial metabolites have been associated with reduced bone mineral density and altered bone metabolic indexes (21). Moreover, microbiome profiles have differed according to circulating levels of procollagen type 1 N-terminal propeptide (P1NP), a marker of bone formation, as well as C-terminal telopeptide of type I collagen (CTX), a marker of bone resorption (22). These findings indicate that the gut microbiota may be related to both sides of bone remodeling, although their observational design does not establish causality or demonstrate a direct effect on osteoblast differentiation.

Human intervention studies provide additional, although inconsistent, evidence. In older women with low bone mineral density, supplementation with Lactobacillus reuteri for 12 months reduced the loss of tibial volumetric bone mineral density compared with placebo (23). Similarly, a combination of three Lactobacillus strains prevented lumbar spine bone loss in early postmenopausal women (24). However, these studies did not demonstrate a consistent increase in circulating bone-formation markers, and a more recent trial found no significant effects of probiotic supplementation on P1NP, bone-specific alkaline phosphatase, osteocalcin, BMD, or fracture risk (25). Therefore, probiotic effects appear to be strain-specific and may vary according to age, skeletal site, baseline bone status, and intervention duration.

The biological mechanisms that may explain these human observations remain largely based on experimental evidence. Microbiota-derived short-chain fatty acids may support bone formation by influencing systemic IGF-1 availability and by regulating immune pathways involved in osteoblast activity. Butyrate has also been proposed to promote a Treg-dependent increase in Wnt10b, a signal involved in osteoblast differentiation. However, these pathways have not yet been directly confirmed in humans, and they should therefore be presented as possible explanations rather than established clinical mechanisms.

Overall, current human evidence supports an association between the gut microbiota, bone-formation markers, and the maintenance of bone density, but does not yet demonstrate a direct causal effect on osteoblast differentiation. The most plausible mechanisms include modulation of microbial metabolites, systemic inflammatory tone, nutrient availability, and endocrine signals such as IGF-1. Further human studies combining microbiome analysis with circulating metabolites, P1NP, osteocalcin, bone-specific alkaline phosphatase, and longitudinal imaging are required to clarify the osteoblast-related component of the gut-bone axis.

### Additional microbiota-related metabolites involved in the gut-bone axis

Besides short-chain fatty acids and menaquinones, other microbiota-related metabolites may contribute to the gut-immune-bone axis. Relevant candidates include secondary bile acids, polyphenol-derived aromatic metabolites, tryptophan-derived indoles, and trimethylamine N-oxide.

Human multi-omics and metabolomic studies provide preliminary evidence that these metabolites may be related to bone health. In peri- and early postmenopausal women, 3-phenylpropanoic acid, mainly derived from microbial metabolism of dietary polyphenols, and glycolithocholic acid, a secondary bile acid, were identified among metabolites potentially related to BMD, although the associations did not remain significant after correction for multiple testing (26). Another metabolomic study identified several circulating metabolites associated with low or high BMD, including bile acids (27). These findings suggest a possible link between microbial transformation of dietary compounds, bile acid metabolism, and bone remodeling, but they remain exploratory and do not prove causality.

Bile acids may influence bone health through fat and fat-soluble vitamin absorption, as well as metabolic and inflammatory regulation. Similarly, microbial metabolism of polyphenols from plant foods produces smaller aromatic compounds that may be more bioavailable, although their specific effects on human bone formation and resorption remain uncertain (26, 27).

Tryptophan-derived indoles may represent another relevant pathway. Gut bacteria can convert tryptophan into indole derivatives, including indole-3-acetic acid and indole-3-propionic acid, which may support intestinal barrier integrity and immune regulation through the aryl hydrocarbon receptor. In an ovariectomized mouse model, these metabolites improved gut barrier function, increased IL-10 signaling, supported osteoblast differentiation, and reduced osteoclastogenesis (28). However, comparable bone-specific effects have not yet been demonstrated in humans.

TMAO may instead represent a potentially unfavorable host-microbial co-metabolite. Gut bacteria convert dietary choline and L-carnitine into trimethylamine, which is then oxidized to TMAO mainly in the liver. Circulating TMAO therefore reflects the interaction between diet, microbiota, hepatic metabolism, and renal clearance (29). Human data linking TMAO with bone outcomes remain inconsistent: one prospective study found no overall association with hip fracture risk or total hip BMD, except for an exploratory association in individuals with overweight or obesity (30), whereas a cross-sectional study in type 2 diabetes associated higher TMAO with lower BMD and higher prevalence of osteoporosis and previous fractures (31).

Overall, secondary bile acids, polyphenol-derived metabolites, tryptophan-derived indoles, and TMAO may represent additional mediators of the gut-bone axis. However, most human evidence remains observational, and several of these compounds are better considered host-microbial co-metabolites rather than purely bacterial products. Their effects are likely to depend on diet, microbial composition, metabolic health, renal function, and inflammatory status.

## Gut dysbiosis, dietary patterns, and immune activation

Gut dysbiosis may impair intestinal barrier integrity and increase systemic exposure to microbial-associated molecular patterns, including lipopolysaccharides and other bacterial components (12). This process may activate innate immune receptors, sustain low-grade inflammation, and amplify cytokine-mediated signaling.

Dietary patterns are among the principal environmental determinants of microbial ecology. Western dietary patterns characterized by high intake of saturated fats, refined carbohydrates, and low fiber availability are associated with reduced microbial diversity, impaired epithelial barrier integrity, and altered microbial metabolism (15, 16).

Reduced intake of fermentable fibers may decrease the production of short-chain fatty acids such as butyrate, acetate, and propionate (16). These metabolites support epithelial barrier integrity and promote regulatory immune pathways, including Treg maintenance (16). Consequently, reduced short-chain fatty acid availability may favor a more pro-inflammatory immune phenotype.

Within the osteo-immune axis, these inflammatory changes are relevant because cytokine-mediated immune activation contributes to osteoclast differentiation and inflammatory bone resorption (4, 13). Thus, dietary patterns may indirectly influence skeletal remodeling through microbial and immune-mediated pathways.

Microbiota-modulating strategies, including prebiotics, probiotics, synbiotics, and postbiotics, may theoretically influence inflammatory tone by altering microbial ecology and metabolite production (12, 16). Experimental evidence suggests that some probiotic strains may modulate cytokine production, macrophage activity, and intestinal barrier function. However, evidence remains heterogeneous, and current clinical data are insufficient to support definitive conclusions regarding skeletal outcomes.

### Dietary modulation of microbiota-mediated immune signaling

Dietary patterns may influence immune regulation through coordinated effects on gut microbial composition, microbial metabolism, intestinal barrier integrity, and host immune responses. Recent reviews summarize the potential role of probiotics, fermented foods, fibers, and fruit-derived bioactives in microbiota-mediated immune modulation (32, 33). Human intervention studies increasingly support this diet-microbiota-immune connection, although the magnitude and direction of the response vary considerably between individuals and dietary exposures.

In a prospective dietary intervention with longitudinal microbiome and immune profiling, increased consumption of fermented foods was associated with greater microbiome diversity and reduced circulating inflammatory markers. By contrast, increased dietary fiber altered microbial functions but produced more individualized immune responses, suggesting that baseline microbiota and microbial capacity to metabolize fiber may influence the effects of dietary intervention (34). In individuals with prediabetes, both a Mediterranean diet and a personalized postprandial glucose-targeting diet modified microbial species and pathways, circulating metabolites, and cytokines. Mediation analyses suggested that selected microbial features may partly contribute to the metabolic and immune responses to diet (35).

Short-term dietary changes may also rapidly influence immune function. A controlled feeding study comparing vegan and ketogenic diets identified distinct changes in microbiome composition, microbial metabolic pathways, immune-cell populations, and immune-related gene expression after only two weeks (36). More recently, a randomized trial comparing Western and African heritage-style dietary patterns showed that a Western dietary transition promoted a pro-inflammatory profile, whereas the plant-rich heritage diet and a traditional fermented beverage produced predominantly anti-inflammatory changes in circulating proteins and cytokine responses (37).

These findings are consistent with the potential immunomodulatory effects of dietary fiber, fermented foods, probiotics, and plant-derived bioactive compounds. Fibers provide substrates for microbial fermentation, whereas polyphenols and other plant compounds are transformed into smaller microbial or host-microbial metabolites. Fermented foods may additionally provide live microorganisms and fermentation-derived compounds. However, these effects should not be generalized across all foods or probiotic products. A recent controlled kombucha intervention produced modest microbiome changes without significant reductions in circulating inflammatory markers, emphasizing the importance of food type, microbial strain, dose, duration, host characteristics, and baseline microbiome composition (38).

**Figure 1:**
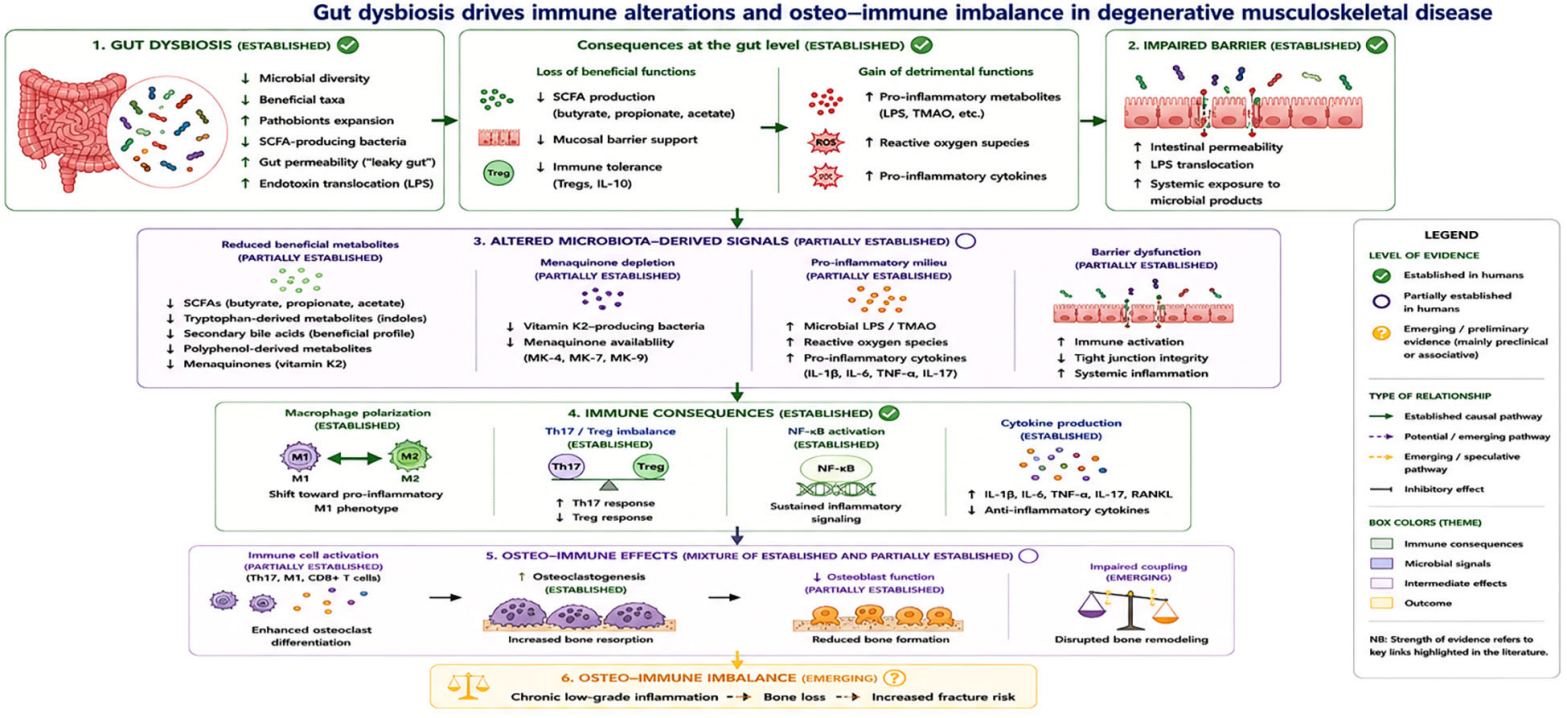
Proposed gut dysbiosis-immune-bone axis in degenerative musculoskeletal disease. Evidence classification: green boxes and solid arrows indicate established mechanisms; purple boxes and dashed arrows indicate partially established mechanisms; yellow boxes and dash-dot arrows indicate emerging or hypothetical pathways requiring further validation. The classification refers to the strength of evidence supporting the main biological links represented in the figure.

From an osteo-immune perspective, dietary modulation of the microbiota may be relevant because intestinal barrier dysfunction, microbial products, oxidative stress, and persistent cytokine production can affect both osteoclast and osteoblast activity. Nevertheless, the current human evidence primarily supports modulation of upstream microbiome and immune pathways rather than a direct causal effect of fermented foods, probiotics, fruits, or fiber on osteo-immune remodeling. The proposed gut dysbiosis–immune–bone framework and the evidence levels supporting its main pathways are summarized in **Figure 1**.

### Vitamin D and menaquinones: comparative immunological activities

To further clarify the proposed framework, the immunological activities of vitamin D and vitamin K2/menaquinones are summarized in **Supplementary Table 1**. This comparison is not intended to suggest that the two vitamin systems have equivalent levels of evidence. Vitamin D has a more established role in immune regulation, particularly through VDR-mediated effects on innate and adaptive immune cells (5–7, 39). In contrast, the immunological role of vitamin K2 and microbiota-derived menaquinones remains less defined and is supported mainly by experimental, indirect, or pathway-based evidence (17–20, 40).

**Figure 2:**
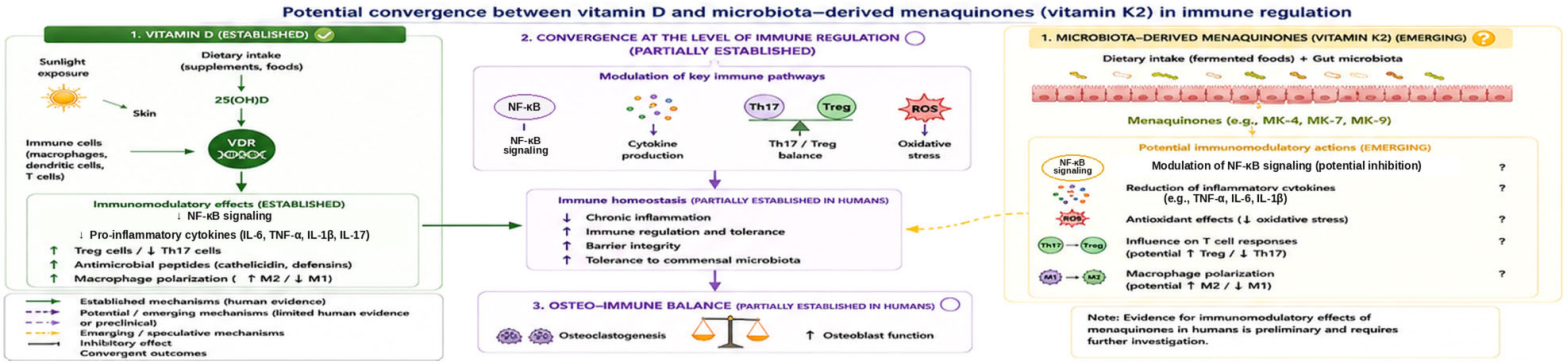
Potential convergence between vitamin D and microbiota-derived menaquinones in immune regulation. Evidence classification: green boxes and solid arrows indicate established mechanisms; purple boxes and dashed arrows indicate partially established mechanisms; yellow boxes and dash-dot arrows indicate emerging or hypothetical pathways requiring further validation. The potential immunoregulatory effects of microbiota-derived menaquinones are classified as emerging because direct human evidence remains limited.

The table therefore distinguishes between established, partially supported, and emerging mechanisms. This distinction is particularly important because vitamin D can be more directly linked to immune-cell function, cytokine regulation, antimicrobial responses, and Th17/Treg balance (5–7, 39). Menaquinones, instead, are better interpreted as potential modulators of inflammatory signaling, vitamin K-dependent proteins, redox balance, ferroptosis, and macrophage-related responses (17–20, 40). The potential convergence between vitamin D and microbiota-derived menaquinones is illustrated in **Figure 2**.

### Dietary vitamin K versus microbiota-derived menaquinones

Dietary vitamin K intake may have a greater and more predictable influence on systemic vitamin K availability than bacterially synthesized menaquinones. From a nutritional perspective, phylloquinone from green leafy vegetables and menaquinones from fermented and animal-derived foods are directly introduced with the diet and absorbed mainly in the small intestine together with dietary fat. By contrast, microbiota-derived menaquinones are mainly produced in the colon, where their absorption and contribution to systemic vitamin K status remain less clearly defined.

This distinction is important when discussing immune regulation. If vitamin K influences immune pathways through vitamin K-dependent proteins, inflammatory signaling, redox balance, or macrophage-related mechanisms, dietary intake is currently the more measurable and clinically modifiable factor (40). In support of this caution, fecal menaquinone concentrations have been shown to be associated with gut microbiota composition, but not clearly with circulating vitamin K concentrations or fecal cytokine concentrations in healthy adults (41). Therefore, bacterially synthesized menaquinones should not be assumed to have the same systemic immunological relevance as dietary vitamin K.

However, microbiota-derived menaquinones should not be dismissed. They may still be relevant within the intestinal ecosystem, where they could influence microbial cross-feeding, local bacterial metabolism, epithelial-immune interactions, and the luminal inflammatory environment. Moreover, dietary vitamin K itself may interact with the gut microbiota: experimental evidence indicates that dietary vitamin K can be remodeled by gut bacteria and may influence microbial community composition (42). Large-scale metagenomic analyses also show that the human gut microbiota possesses variable genetic potential for vitamin biosynthesis, including menaquinone production, depending on host age, geography, lifestyle, and microbial composition (43).

Thus, dietary vitamin K and microbiota-derived menaquinones should be viewed as interacting but not equivalent layers of the proposed framework. Dietary vitamin K probably has the greater influence on systemic vitamin K-dependent immune pathways, whereas bacterially synthesized menaquinones may represent a more local, context-dependent, and still emerging component of the gut-immune-bone axis. Future studies should distinguish dietary intake, circulating vitamin K status, fecal menaquinone profiles, microbial vitamin biosynthetic capacity, and immune outcomes to clarify their relative contributions.

### Context-dependent modifiers: age, antibiotics, and chronic metabolic disease

The proposed gut-immune-bone interactions are likely to vary according to host context. Age, antibiotic exposure, and chronic metabolic diseases may modify the same biological network by altering microbial diversity, intestinal barrier integrity, immune tone, microbial metabolite production, and bone-cell responsiveness to inflammatory signals.

Ageing may amplify the skeletal consequences of gut dysbiosis. With age, immunosenescence and chronic low-grade inflammation become more prominent, while bone remodeling becomes less efficient because bone formation declines and inflammatory cytokines such as TNF-α, IL-1, and IL-6 may promote osteoclast activity (44). In this context, dysbiosis may have stronger skeletal effects than in younger individuals. Human prospective data support the clinical relevance of this interaction: in the FINRISK cohort, higher gut microbial alpha diversity was associated with lower fracture risk, whereas higher Proteobacteria, particularly Gammaproteobacteria, was associated with increased fracture risk (45). These data do not prove causality, but suggest that microbiota composition may be related to long-term skeletal vulnerability.

Antibiotic exposure may act as an acute or repeated ecological perturbation of the gut-immune-bone axis. In healthy adults, commonly used antibiotics can reduce microbial richness and alter microbial composition, resistome, and metabolic output, with some changes persisting for months (46). Experimental evidence suggests that post-antibiotic dysbiosis can induce immune-dependent trabecular bone loss (47). Human observational data are consistent with this possibility: in a large Korean cohort of adults aged 50 years or older, long-term antibiotic exposure and exposure to multiple antibiotic classes were associated with higher osteoporotic fracture risk (48). However, these findings should be interpreted cautiously because antibiotic use may also reflect infections, comorbidities, frailty, medication use, or other confounders.

Chronic metabolic diseases may further shift the axis toward inflammation-driven bone remodeling. Obesity and type 2 diabetes are commonly associated with low-grade inflammation, altered gut permeability, changes in microbial metabolites, insulin resistance, oxidative stress, and increased exposure to bacterial products such as lipopolysaccharide (49). These signals may favor macrophage activation, cytokine production, Th17/Treg imbalance, and osteoclastogenic signaling, while impairing osteoblast activity and bone quality. Experimental evidence also suggests that obesity-associated gut microbiota may contribute to skeletal deterioration through bone marrow macrophage senescence and grancalcin secretion (50). In diabetes, hyperglycemia, advanced glycation end products, oxidative stress, and chronic inflammation can impair bone quality even when BMD is not markedly reduced (51).

Overall, age, antibiotics, and chronic metabolic disease should be considered context-dependent modifiers rather than isolated variables. Age may reduce immune and skeletal resilience; antibiotics may disrupt microbial ecology and metabolite production; and metabolic disease may amplify dysbiosis-related inflammatory signaling. Future studies should therefore stratify participants by age, antibiotic exposure, metabolic status, medication use, dietary pattern, and baseline microbiota composition when testing the gut-immune-bone framework.

### Candidate biomarkers for clinical validation of the gut-immune-bone axis

The proposed gut-immune-bone axis could be tested in clinical studies using an integrated biomarker panel including microbiota composition, microbial metabolites, intestinal barrier and endotoxin-related markers, vitamin D and vitamin K status, mineral metabolism, bone turnover, and skeletal outcomes. From a nutritional perspective, this approach allows the model to be evaluated through measurable diet-sensitive pathways rather than through a single immune or bone marker. The candidate biomarkers and their practical interpretation are summarized in **Supplementary Table 2**.

Stool-based microbiota analyses could include alpha diversity indexes, such as Shannon diversity, richness, and Pielou evenness, together with beta diversity, Proteobacteria/Gammaproteobacteria abundance, SCFA-producing taxa, and metagenomic pathways related to butyrate, menaquinone, and LPS biosynthesis (45). Metabolic assessment could include fecal and/or circulating SCFAs, TMAO, secondary bile acids, tryptophan-derived indoles, polyphenol-derived aromatic metabolites, and fecal or circulating menaquinone profiles (26–31).

Vitamin and mineral assessment should include serum 25(OH)D, PTH, calcium, phosphate, magnesium, renal function, dietary vitamin K intake, undercarboxylated osteocalcin, dephosphorylated-uncarboxylated matrix Gla protein, and, when technically available, fecal and circulating menaquinones (5–7, 9, 39–43). These markers may help distinguish the more measurable contribution of dietary vitamin K from the less certain systemic role of microbiota-derived menaquinones.

Bone remodeling should be assessed using standardized markers and imaging outcomes. Serum P1NP and plasma β-CTX-I are recommended reference markers of bone formation and resorption, respectively (52). Osteocalcin and bone-specific alkaline phosphatase may provide complementary information, while DXA-derived BMD, trabecular bone score, and fracture incidence can assess skeletal outcomes.

Markers of intestinal function and endotoxin exposure may include fecal calprotectin, fecal secretory IgA, stool pH, circulating LPS, LPS-binding protein, soluble CD14, and intestinal fatty acid-binding protein (53, 54). Zonulin and urinary indican could be considered exploratory markers of intestinal permeability or proteolytic dysbiosis, although their interpretation requires caution because of limited standardization and variable clinical validity, particularly for commercially available zonulin assays (55).

Finally, mechanistic studies may include RANKL, OPG, the RANKL/OPG ratio, inflammatory cytokines, and Th17/Treg balance. However, these should be considered optional research markers rather than core clinical biomarkers, because circulating immune and osteo-immune markers may not fully reflect the local bone microenvironment.

Overall, clinical validation should prioritize a feasible core panel including stool microbiota analysis, microbial metabolites, intestinal and endotoxin-related markers, vitamin D and vitamin K status, mineral metabolism, bone turnover markers, and DXA-derived outcomes, with advanced immune markers added in translational or mechanistic studies.

## Integrated hypothesis

This Perspective proposes a mechanistic framework in which gut dysbiosis may contribute to degenerative musculoskeletal and metabolic bone diseases through alterations in vitamin-dependent immune regulation.

Within this model, dysbiosis acts as one potential amplifier of inflammatory signaling. Altered microbial composition, reduced diversity, impaired barrier integrity, and increased exposure to microbial-associated molecular patterns may promote activation of innate and adaptive immune pathways (12). At the same time, impaired vitamin D signaling may reduce immunoregulatory control over macrophage activation, dendritic cell maturation, and T-cell polarization (5–7). Concurrently, alterations in microbiota-derived menaquinone production may influence inflammatory signaling pathways associated with NF-κB activation and cytokine regulation (8–11, 17–20).

Together, these processes could theoretically contribute to a pro-inflammatory cytokine environment enriched in IL-6, TNF-α, IL-17, and IL-1β (4, 13). This inflammatory milieu may favor osteoclastogenesis through the RANKL/RANK/OPG axis, impair osteoblast activity, and progressively disrupt bone remodeling.

Importantly, this model does not interpret vitamin D, menaquinones, or nutritional factors as isolated determinants of skeletal degeneration. Rather, these factors are conceptualized as modulators of immune tone integrated within a broader osteo-immune network.

### Longitudinal studies to test the proposed framework

Longitudinal cohort studies could represent a realistic first step to test the proposed gut-immune-bone framework in humans. From a nutritional and clinical perspective, the aim would not be to prove a single causal mechanism, but to evaluate whether changes in diet-sensitive microbiome and metabolic markers precede or accompany changes in immune activation and bone remodeling.

Suitable populations may include peri- or postmenopausal women, older adults, or individuals with chronic metabolic diseases. At baseline and during follow-up, participants should be characterized for dietary intake, vitamin D and vitamin K intake, physical activity, body composition, medication use, antibiotic exposure, renal function, metabolic health, and inflammatory status, as these factors may influence both microbiota and bone outcomes.

Repeated stool and blood assessments could include microbiota diversity, SCFA-producing taxa, Proteobacteria abundance, and functional pathways related to butyrate, menaquinone, and LPS biosynthesis. Metabolite profiling could include SCFAs, TMAO, secondary bile acids, tryptophan-derived indoles, polyphenol-derived metabolites, and fecal or circulating menaquinones. These data could be integrated with intestinal and endotoxin-related markers, vitamin D and vitamin K status, PTH, calcium, phosphate, bone turnover markers such as P1NP and β-CTX-I, and DXA-derived BMD.

More advanced studies could add immune phenotyping, including cytokines, Th17/Treg balance, RANKL, OPG, and the RANKL/OPG ratio. This would allow researchers to explore whether microbial metabolites or immune activation mediate the relationship between dysbiosis and bone remodeling.

However, longitudinal cohorts would still remain observational. They could strengthen temporal plausibility and identify candidate microbiome-immune-bone signatures, but they would not definitively prove causality. For this reason, a stepwise approach may be preferable: longitudinal studies to identify reproducible associations, followed by targeted dietary or microbiota-modulating interventions to test whether modifying these pathways affects bone turnover and skeletal outcomes.

## Discussion

The present Perspective integrates microbial ecology, inflammatory signaling, and vitamin-dependent immune pathways into an immunology-centered framework of skeletal degeneration. Rather than interpreting degenerative musculoskeletal disease exclusively through endocrine or structural paradigms, this model emphasizes the role of chronic immune dysregulation and osteo-immune imbalance (4, 14).

The strongest component of the proposed framework is represented by vitamin D-dependent immune regulation. The immunomodulatory effects of vitamin D on macrophages, dendritic cells, cytokine production, and T-cell differentiation are supported by substantial mechanistic evidence (5–7). In contrast, the proposed immunomodulatory role of microbiota-derived menaquinones should currently be interpreted primarily as a hypothesis-generating concept supported mainly by preclinical and indirect evidence (8–11, 17–20, 40).

Nevertheless, the inclusion of menaquinones within the gut-immune-bone axis may provide an interesting conceptual advance. Positioning microbiota-derived menaquinones as modulators of inflammatory tone rather than direct skeletal regulators may help integrate nutritional, microbial, and immunological perspectives without oversimplification.

Several limitations must be acknowledged. Causal pathways linking dysbiosis, vitamin-dependent signaling, and skeletal outcomes remain difficult to disentangle because endocrine, metabolic, nutritional, and lifestyle variables strongly interact. Much of the available evidence derives from experimental or observational studies rather than mechanistic human trials. Furthermore, the contribution of bacterially produced menaquinones to systemic immune physiology in humans remains incompletely defined.

Additional conceptual and methodological uncertainties also remain. Fecal microbiota composition may not accurately reflect mucosa-associated microbial activity, and distinguishing causal microbial alterations from secondary changes associated with aging, diet, medication use, antibiotic exposure, or chronic disease remains challenging.

Future research should aim to integrate microbiome profiling, immune phenotyping, cytokine analysis, vitamin pathway assessment, biomarkers of microbial metabolites and endotoxin exposure, and skeletal outcomes within longitudinal human studies. Particular attention should be directed toward understanding how microbial vitamin metabolism influences immune cell function and inflammatory signaling.

Overall, this Perspective supports the idea that degenerative musculoskeletal and metabolic bone diseases may partly emerge from persistent immune dysregulation sustained by microbial and vitamin-dependent pathways. Within this context, microbiota-derived menaquinones may represent an underexplored component of osteoimmune biology deserving further mechanistic investigation.

## Data Availability

The original contributions presented in the study are included in the article/**Supplementary Material**. Further inquiries can be directed to the corresponding author.
