# Supplementary material for: Gut dysbiosis and vitamin-dependent immune regulation in degenerative musculoskeletal and bone diseases
Source: Front Immunol. 2026 Jul 30;17:1920962. doi: 10.3389/fimmu.2026.1920962 (PMC13468887; doi:10.3389/fimmu.2026.1920962)
Supplement: Supplementary file 1 [file DataSheet1.pdf]

Supplementary material

Supplementary Table 1. Comparative immunological activities of vitamin D and vitamin K2/menaquinones relevant to the gut-immune-bone axis

Domain	Vitamin D	Vitamin K2 / menaquinones	Interpretation
Main source	Sunlight exposure, diet, and supplements; converted to 25(OH)D and active vitamin D metabolites.	Diet, fermented foods, animal foods, and possible bacterial synthesis in the gut.	Vitamin D has a clearer endocrine framework. Microbiota-derived menaquinones remain harder to quantify in humans.
Immune target	VDR is expressed by several immune cells, including macrophages, dendritic cells, and T cells [5-7,39].	Potential effects may involve vitamin K-dependent proteins, macrophages, inflammatory signaling, and redox pathways [17-20,40].	Vitamin D has stronger direct evidence in immune regulation.
Innate immunity	Supports antimicrobial responses and modulates monocytes, macrophages, and dendritic cells [5-7,39].	May influence innate immunity through NF-κB modulation, Gas6/Protein S-TAM signaling, and macrophage responses [17-20,40].	Vitamin D = more established; K2/menaquinones = plausible but less established.
Inflammatory cytokines	Generally associated with modulation of pro-inflammatory signaling, including IL-6, TNF-α, IL-1β, and IL-17 [5-7,39].	Experimental evidence suggests possible reduction of inflammatory mediators, depending on vitamer, side-chain length, dose, and model [19,40].	Both may converge on inflammatory tone, but evidence is stronger for vitamin D.
T-cell regulation	May reduce excessive Th1/Th17 responses and support regulatory T-cell activity [5-7,39].	Possible effects on T-cell responses are mostly indirect, through inflammation control, macrophage-related pathways, or redox regulation [19,40].	Direct evidence for Th17/Treg modulation is stronger for vitamin D.
Macrophage activity and inflammatory resolution	May support a less inflammatory macrophage phenotype and modulate antigen-presenting cell function [5-7,39].	Vitamin K-dependent proteins such as Gas6 and Protein S may interact with TAM receptors, which are involved in macrophage activity, apoptotic cell clearance, and resolution of inflammation [17,40].	Useful mechanistic bridge for vitamin K biology, but direct evidence for microbiota-derived menaquinones remains limited.
Oxidative stress and ferroptosis	May indirectly reduce oxidative stress through immune and metabolic regulation [5-7,39].	Reduced vitamin K forms may limit lipid peroxidation and ferroptosis through the FSP1-dependent vitamin K cycle [18,40].	K2/menaquinones provide a pathway beyond NF-κB, but clinical relevance to bone-immune remodeling is uncertain.
Bone-immune interface	Links calcium-phosphate metabolism with immune regulation and bone remodeling [5-7,39].	Links γ-carboxylation of bone-related proteins with possible immune and redox effects [17-20,40].	Vitamin D is more established; K2 is promising but should be presented as emerging.
Evidence level in this framework	Established / partially established, depending on the specific pathway.	Emerging, especially when referring specifically to microbiota-derived menaquinones.	The two vitamins should not be presented as equivalent in evidence strength.

Supplementary Table 2. Candidate biomarkers for clinical validation of the gut-immune-bone axis

Layer	Candidate biomarkers	Practical interpretation
Gut microbiota composition	Shannon index, richness, Pielou evenness, beta diversity, Proteobacteria/Gammaproteobacteria, SCFA-producing taxa	To assess dysbiosis, diversity loss, and potentially pro-inflammatory microbial patterns
Microbial functional potential	Metagenomic pathways for butyrate synthesis, menaquinone biosynthesis, LPS biosynthesis	To evaluate whether microbial function, not only composition, is related to immune and bone outcomes
Microbial metabolites	Fecal/circulating acetate, propionate, butyrate; TMAO; secondary bile acids; tryptophan-derived indoles; polyphenol-derived aromatic metabolites; fecal/circulating menaquinones	To test whether microbial metabolic activity mediates the gut-immune-bone relationship
Stool / gut function markers	Fecal calprotectin, fecal secretory IgA, stool pH, digestive/fermentative markers	To assess gut inflammatory status, mucosal immune activity, and intestinal ecosystem function
Endotoxin exposure / microbial translocation	Circulating LPS, LPS-binding protein, soluble CD14, intestinal fatty acid-binding protein	To explore whether dysbiosis and barrier dysfunction are associated with systemic exposure to bacterial products
Exploratory permeability / dysbiosis markers	Zonulin, urinary indican	To explore gut barrier dysfunction or proteolytic dysbiosis, with cautious interpretation
Vitamin D-mineral axis	25(OH)D, PTH, calcium, phosphate, magnesium, renal function	To evaluate clinically modifiable endocrine and mineral contributors to bone remodeling
Vitamin K status	Dietary vitamin K intake, undercarboxylated osteocalcin, dp-ucMGP, fecal menaquinones, circulating menaquinones	To distinguish dietary vitamin K status from bacterial menaquinone production and systemic availability
Bone turnover	P1NP, β-CTX-I, osteocalcin, bone-specific alkaline phosphatase	To evaluate bone formation and resorption dynamics
Bone outcomes	DXA-BMD, trabecular bone score, fracture incidence	To assess whether biomarker patterns translate into clinically relevant skeletal outcomes
Optional osteo-immune markers	RANKL, OPG, RANKL/OPG ratio	To explore osteoclastogenic signaling in mechanistic studies
Optional immune markers	hs-CRP, IL-6, TNF-α, IL-1β, IL-17A, IL-10, Th17/Treg ratio	To evaluate systemic inflammatory tone and immune polarization in research settings
